# m^6^A modification of AC026356.1 facilitates hepatocellular carcinoma progression by regulating the IGF2BP1-IL11 axis

**DOI:** 10.1038/s41598-023-45449-w

**Published:** 2023-11-05

**Authors:** Huamei Wei, Jinhun Yang, Rongzhou Lu, Yanyan Huang, Zheng Huang, Lizheng Huang, Min Zeng, Yunyu Wei, Zuoming Xu, Wenchuan Li, Jian Pu

**Affiliations:** 1 Clinical Pathological Diagnosis and Research Centre, Affiliated Hospital of Youjiang Medical University for Nationalities, Baise, China; 2 Graduate College of Youjiang Medical University for Nationalities, Baise, China; 3 Department of Hepatobiliary Surgery, Affiliated Hospital of Youjiang Medical University for Nationalities, No. 18 Zhongshan Two Road, Baise, 533000 China; 4 Guangxi Clinical Medical Research Center of Hepatobiliary Diseases, Baise, China

**Keywords:** RNA, Hepatocellular carcinoma

## Abstract

N^6^-methyladenosine (m^6^A) is the most common RNA modification in eukaryotic RNAs. Although the important roles of m^6^A in RNA fate have been revealed, the potential contribution of m^6^A to RNA function in various diseases, including hepatocellular carcinoma (HCC), is still unclear. In this study, we identified a novel m^6^A-modified RNA AC026356.1. We found that AC026356.1 was increased in HCC tissues and cell lines. High expression of AC026356.1 was correlated with poor survival of HCC patients. m^6^A modification level of AC026356.1 was also increased in HCC and more significantly correlated with poor survival of HCC patients. Functional assays showed that m^6^A-modified AC026356.1 promoted HCC cellular proliferation, migration, and liver metastasis. Gene set enrichment analysis showed that AC026356.1 activated IL11/STAT3 signaling. Mechanistic investigation showed that m^6^A-modified AC026356.1 bound to IGF2BP1. The interaction between m^6^A-modified AC026356.1 and IGF2BP1 promoted the binding of IL11 mRNA to IGF2BP1, leading to increased IL11 mRNA stability and IL11 secretion. Functional rescue assays showed that depletion of IL11 reversed the oncogenic roles of AC026356.1. These findings revealed the potential influences of m^6^A modification on RNA biological functions and suggested that targeting m^6^A modification may be a novel strategy for HCC treatment.

## Introduction

Hepatocellular carcinoma (HCC), the major type of primary liver cancer, is the six most common and the third deadliest malignancy globally^1^. Many of HCC patients are diagnosed at late stages, who are ineligible for surgical resection^2^. Moreover, about 50% of HCC patients recur after surgery^3^. Unfortunately, HCC are resistant against conventional chemotherapies^4^. Although recently developed molecule targeted therapies and immunotherapies have been approved for the treatment of advanced HCC, only a small ratio of HCC patients has clinical benefit from these therapies^5–7^. Thus, further elucidating the molecular mechanisms underlying the oncogenic processes of HCC are urgently needed to develop more effective therapies.

Diverse genomic alterations have been characterized in HCC, including the mutations, amplifications, or depletions of *TERT*, *TP53*, *MYC*, *CDKN2A*, *CTNNB1*, *AXIN1*, *JAK1*, et al.^8^. Apart from genomic alterations, dysregulated expressions of various oncogenes and tumor suppressors may be caused by aberrant epigenetic modifications, including DNA methylation, histone methylation, histone acetylation, long non-coding RNAs (lncRNAs), et al.^9–11^. RNA modification is another layer of gene expression modulation, which is named as epitranscriptomic regulation^12,13^. N^6^-methyladenosine (m^6^A) is the most abundant internal modification in eukaryotic mRNAs^14^. m^6^A modification is a reversible process, which is installed by m^6^A methyltransferases (writers) and removed by m^6^A demethylases (erasers)^15,16^. m^6^A-modified RNAs are recognized by m^6^A readers. Increasing evidences have shown that m^6^A modification plays critical roles in RNA fate and function, such as RNA splicing, export, stability, and translation^16–19^. Thus, through regulating RNA fate, m^6^A modification participates in various physiopathological processes^20–22^. Consistently, m^6^A modification of different mRNAs show diverse roles in malignancies, including HCC^23,24^. m^6^A modification increased ANLN mRNA stability, which enhanced the roles of ANLN in promoting HCC bone metastasis^25^. m^6^A modification induced SOCS2 mRNA degradation, which abolished the tumor suppressive roles of SOCS2 in liver cancer^26^. Apart from mRNAs, several lncRNAs have been revealed to be m^6^A modified^27^. Similarly, m^6^A modification may change the fate of lncRNAs^28^. However, the potential influences of m^6^A modification on lncRNAs functions in HCC are still largely unknown.

Through causing aberrant expression or function of target molecules, these genomic alterations, epigenetic aberrations, and aberrant m^6^A modification may further cause signaling pathways deregulation in HCC^29–31^. The critical HCC-related signaling pathways include WNT–β-catenin signaling, PI3K-Akt signaling, mTOR signaling, JAK-STAT signaling, TGF-β signaling, et al.^32,33^. Signal transducer and activator of transcription 3 (STAT3) is a critical member of the JAK-STAT signaling^34^. In response to cytokines or growth factors, such as IL6, IL10, IL11, EGF, HGF, BMP2, STAT3 is phosphorylated and translocates to nucleus where it functions as transcription activators and regulates downstream signaling^35,36^. STAT3 signaling plays critical oncogenic roles in HCC, such as promoting HCC cellular proliferation and survival^37,38^.

We investigated the m^6^A-related lncRNAs in HCC through analyzing The Cancer Genome Atlas (TCGA) Liver hepatocellular carcinoma (LIHC) database. Among these previously reported m^6^A-related lncRNAs in HCC^39^, we further found that the m^6^A modification levels of some of these lncRNAs are also dysregulated in HCC. Here, we report one of these lncRNAs, AC026356.1. Not only the expression level, but also the m^6^A modification level of AC026356.1 is increased in HCC and correlated with poor prognosis. Furthermore, we found that only m^6^A-modified AC026356.1, but not non m^6^A-modified AC026356.1, had oncogenic roles in HCC.

## Materials and methods

### Tissue samples

Gene expression values derived from TCGA LIHC data were download from https://portal.gdc.cancer.gov/. The correlation between gene expression and survival derived from TCGA LIHC data was calculated by the on line tool GEPIA (Gene Expression Profiling Interactive Analysis, http://gepia.cancer-pku.cn/)^40^. We randomly collected 65 pairs of HCC tissues and matched noncancerous liver tissues from our hospital. Written informed consents were signed by all HCC patients. The use of tissue samples followed the Declaration of Helsinki. The Affiliated Hospital of Youjiang Medical University for Nationalities Institutional Review Board reviewed and approved this study.

### Cell culture and treatment

Human immortalized liver cell line THLE-2 (cat. no. CRL-2706) and HCC cell line SNU-398 (cat. no. CRL-2233) were acquired from American Type Culture Collection (ATCC, Manassas, VA, USA). Human HCC cell lines HuH-7 (cat. no. SCSP-526) and Hep3B (cat. no. SCSP-5045) were acquired from the Chinese Academy of Sciences Cell Bank (Shanghai, China). Human HCC cell line HCCLM3 (cat. no. C6303) was acquired from Beyotime Biotechnology (Shanghai, China). Cells were cultured strictly following the provided protocols in a humidified incubator at 37 °C with 5% CO_2_. For RNA degradation assay, indicted cells were treated with 50 µM α-amanitin (Sigma-Aldrich, Saint Louis, MO, USA) for indicated time as in the figures. All cells were routinely tested as mycoplasma-free using the Mycoplasma Stain Assay Kit (cat. no. C0296, Beyotime).

### RNA isolation, reverse transcription, and quantitative polymerase chain reaction (qPCR)

Total RNA was extracted from indicated tissues and cells using the RNA isolater Total RNA Extraction Reagent (cat. no. R401, Vazyme, Nanjing, China). Reverse transcription was performed using the RNA and the HiScript III RT SuperMix for qPCR (cat. no. R323, Vazyme). qPCR was conducted using the ChamQ Universal SYBR qPCR Master Mix (cat. no. Q711, Vazyme) on the QuantStudio 3 Real-Time PCR System (cat. no. A28567, Applied biosystems, Foster City, CA, USA) with the primers: for AC026356.1, 5'-ACAAGGAGCCCATAAACCA-3' (sense) and 5'-AGCAGCAGCCACTACAGAG-3' (antisense); for IL11, 5'-AGGTGGCTCTTCCCTGAA-3' (sense) and 5'-GGGTCACAGCCGAGTCTT-3' (antisense); for IGF2BP1, 5'-GGAAAAACGGTGAACGAGT-3' (sense) and 5'-CTGTCCCTTCTGATGCTG-3' (antisense); for GAPDH, 5'-GTCGGAGTCAACGGATTTG-3' (sense) and 5'-TGGGTGGAATCATATTGGAA-3' (antisense). GAPDH served as internal control. Relative expression was quantified using 2^−ΔΔCt^ method.

### RNA immunoprecipitation (RIP) and methylated RNA immunoprecipitation (MeRIP) assays

To enrich m^6^A-modified transcripts, MeRIP assays were performed in indicated tissues and cells using the Magna MeRIP m^6^A Kit (cat. no. 17-10,499, Millipore, Billerica, MA, USA). Enriched transcripts were subjected to qPCR to detect m^6^A modification level. To detect the RNAs bound to IGF2BP1, RIP assays were performed in indicated cells using the EZ-Magna RIP Kit (cat. no. 17-701, Millipore) and IGF2BP1 specific antibody (cat. no. 712138, Invitrogen, Carlsbad, CA, USA). Enriched RNAs were subjected to qPCR to measure the transcripts bound by IGF2BP1.

### Plasmids, siRNAs, and transfection

The cDNA encoding AC026356.1 was PCR-amplified with the PrimeSTAR Max DNA Polymerase (cat. no. R045Q, Takara, Shiga, Japan) and the primers 5'-GAGACCCAAGCTGGCTAGCCATATGTATAACAAGGCTTTTG-3' (sense) and 5'-GGTTTAAACGGGCCCTCTAGATAGCAACATGGAAAAGCTT-3' (antisense), followed by being subcloned into the *Nhe* I and *Xba* I sites of pcDNA3.1(+) (Invitrogen) with the NovoRec plus One step PCR Cloning Kit (cat. no. NR005, Novoprotein, Shanghai, China). The constructed plasmid or empty pcDNA3.1 plasmid was transfected into SNU-398 and HCCLM3 cells using the GP-transfect-Mate (cat. no. G04009, GenePharma, Shanghai, China) to construct AC026356.1 stably overexpressed or control cells after treatment with 800 µg/ml G418 (cat. no. ant-gn-1, InvivoGen, San Diego, CA, USA) for four weeks. Two pairs of cDNA oligonucleotides targeting AC026356.1 were synthesized and subcloned into the shRNA lentivirus expressing plasmid (GenePharma), which was used to generate shRNA lentivirus targeting AC026356.1. Scrambled non-targeting shRNA lentivirus were used as negative control (NC). The sequences of shRNA oligonucleotides were: for shRNA-AC026356.1-1, 5'-GATCCGGTTAATGCTTACCAACATGTTTCAAGAGAACATGTTGGTAAGCATTAACCTTTTTTG-3' (sense) and 5'-AATTCAAAAAAGGTTAATGCTTACCAACATGTTCTCTTGAAACATGTTGGTAAGCATTAACCG-3' (antisense); for shRNA-AC026356.1-2, 5'-GATCCGCCTTTGGATCTCTAATACTTTTCAAGAGAAAGTATTAGAGATCCAAAGGCTTTTTTG-3' (sense) and 5'-AATTCAAAAAAGCCTTTGGATCTCTAATACTTTCTCTTGAAAAGTATTAGAGATCCAAAGGCG-3' (anti-sense); for shRNA-NC, 5'-GATCCGTTCTCCGAACGTGTCACGTTTCAAGAGAACGTGACACGTTCGGAGAACTTTTTTG-3' (sense) and 5'-AATTCAAAAAAGTTCTCCGAACGTGTCACGTTCTCTTGAAACGTGACACGTTCGGAGAACG-3' (antisense). These shRNA lentiviruses were infected into SNU-398 and HCCLM3 cells to construct AC026356.1 stably silenced or control cells after treatment with 2 µg/ml puromycin (cat. no. ant-pr-1, InvivoGen) for 4 weeks.

WTAP and METTL16 expression and control plasmids were purchased from GenePharma. ON-TARGETplus Human WTAP siRNA SMART Pool (cat. no. L-017323-00-0010), ON-TARGETplus Human METTL16 siRNA SMART Pool (cat. no. L-016359-02-0010), ON-TARGETplus Human IGF2BP1 siRNA SMART Pool (cat. no. L-003977-00-0010), and ON-TARGETplus Human IL11 siRNA SMART Pool (cat. no. L-007927-00-0010) were purchased from Horizon Discovery (Cambridge, England). Cellular transfection of plasmids and siRNAs was conducted using the GP-transfect-Mate (cat. no. G04009, GenePharma).

### Cell proliferation assays

Cell proliferation was assessed using Cell Counting Kit (CCK)-8 and 5-ethynyl-2'-deoxyuridine (EdU) incorporation assays. For CCK-8 assay, indicated cells were plated into 96-well plates at 2000 cells/well and cultured in complete medium. At indicated time, CCK-8 reagent (cat. no. C0042, Beyotime) was added to the plate. After culture for another 2 h, the Synergy 2 microplate reader (BioTek, Winooski, VT, USA) was used to measure the absorbance values at 450 nm. For EdU incorporation assay, indicated cells were seeded into 24-well plates at 10,000 cells/well and cultured overnight. EdU incorporation assay was conducted using the Cell-Light EdU Apollo567 In Vitro Kit (cat. no. C10310-1, RiboBio, Guangzhou, China) as we previously described^41–43^. DAPI was used to stain the nuclei.

### Transwell cell migration assay

Indicated cells suspended in serum free medium were plated into the upper chamber of 24-well transwell inserts (8-μm pore size, BD Biosciences, San Jose, CA, USA) at 50,000 cells/well. Medium containing 20% bovine fetal serum was added to the lower chamber. After culture for 48 h, the cells remaining in the upper chamber were removed and the cells on the lower surface were fixed, stained, and counted using the Imager.M2 fluorescence microscope (Carl Zeiss, Oberkochen, Germany).

### Liver metastasis assay

Indicated HCC cells were intrasplenically injected into 5-week-old male BALB/C athymic nude mice (SpePharm Biotechnology, Beijing, China). After being fed in specific pathogen free condition for 35 days, the mice were euthanized and the livers were resected and subjected to HE staining. The number and diameter of liver metastatic nodules were measured. The animal experiments were performed in accordance with the Animal Research: Reporting of In Vivo Experiments (ARRIVE) guidelines and approved by the Affiliated Hospital of Youjiang Medical University for Nationalities Institutional Review Board. All methods were performed in accordance with the relevant guidelines and regulations.

### IL11 enzyme linked immunosorbent assay (ELISA)

The culture supernatants were collected for 48 h from indicated cells. IL11 concentration in the culture supernatants was measured using the IL-11 Human ELISA Kit (cat. no. EHIL11, Invitrogen) following the provided protocol.

### Statistical analysis

All statistical analyses were conducted using the GraphPad Prism 6.0 Software. Mann–Whitney test, Wilcoxon matched-pairs signed rank test, log-rank test, one-way ANOVA followed by Dunnett's multiple comparisons test, Student’s *t*-test, or Spearman correlation analysis were performed as indicated in figure legends. *P* < 0.05 was considered statistically significant.

## Results

### AC026356.1 is increased and correlated with poor prognosis in HCC

**Figure 1:**
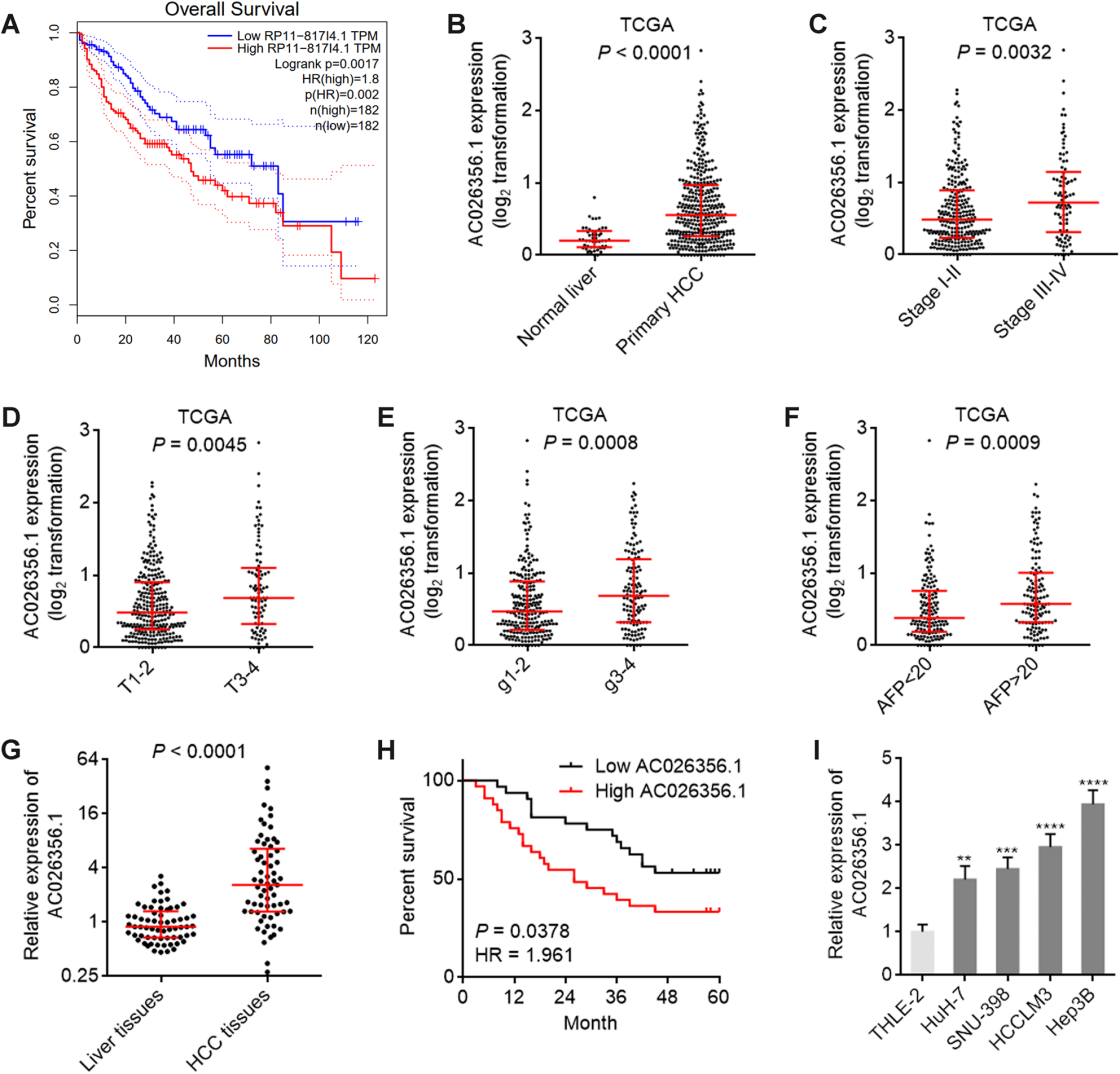
The expression of AC026356.1 in HCC and its correlation with prognosis. (**A**) The correlation between AC026356.1 (RP11-817I4.1) expression and overall survival based on TCGA LIHC data, analysed by the online tool GEPIA. (**B**) AC026356.1 expression in primary HCC tissues (n = 371) and normal liver tissues (n = 50) based on TCGA LIHC data. (**C**) AC026356.1 expression in HCC tissues with pathological stage I-II (n = 257) or III-IV (n = 90) based on TCGA LIHC data. (**D**) AC026356.1 expression in HCC tissues with pathological T 1–2 (n = 275) or 3–4 (n = 93) based on TCGA LIHC data. (**E**) AC026356.1 expression in HCC tissues with grade 1–2 (n = 232) or 3–4 (n = 134) based on TCGA LIHC data. (**F**) AC026356.1 expression in HCC tissues with fetoprotein (AFP) value < 20 (n = 147) or > 20 (n = 131) based on TCGA LIHC data. For (**B**–**F**), data are shown as median with interquartile range. *P* values were calculated using Mann–Whitney test. (**G**) AC026356.1 expression in 65 pairs of HCC tissues and matched noncancerous liver tissues was measured by qPCR. Data are shown as median with interquartile range. *P* values were calculated using Wilcoxon matched-pairs signed rank test. (**H**) Kaplan–Meier analysis of the correlation between AC026356.1 expression and overall survival in HCC (n = 65). Median AC026356.1 expression value was used as cut-off. HR and *P* values were calculated using log-rank test. (**I**) AC026356.1 expression in immortalized liver cell line THLE-2 and HCC cell lines HuH-7, SNU-398, HCCLM3, and Hep3B was measured by qPCR. Data are shown as mean ± standard deviation (SD) of three independent experiments. **P < 0.01, ***P < 0.001, ****P < 0.0001 by one-way ANOVA followed by Dunnett's multiple comparisons test.

Analysis of TCGA LIHC data by the online tool GEPIA (Gene Expression Profiling Interactive Analysis, http://gepia.cancer-pku.cn/) revealed that high expression of AC026356.1 was correlated with poor overall survival (Fig. 1A). Analysis of TCGA LIHC data further revealed that AC026356.1 was increased in HCC tissues compared with normal liver tissues (Fig. 1B). Increased expression of AC026356.1 was positively correlated with pathological stage, pathological T, grade, and fetoprotein (AFP) (Fig. 1C–F). To confirm the expression and clinical relevance of AC026356.1 in HCC, we collected 65 pairs of HCC tissues and matched noncancerous liver tissues, and found that AC026356.1 was also increased in HCC tissues in our cohort (Fig. 1G). High expression of AC026356.1 was also correlated with poor overall survival in our HCC cohort (Fig. 1H). Compared with immortalized human liver cell line THLE-2, AC026356.1 was also increased in HCC cell lines HuH-7, SNU-398, HCCLM3, and Hep3B (Fig. 1I).

### m^6^A modification level of AC026356.1 is increased and correlated with poor prognosis in HCC

**Figure 2:**
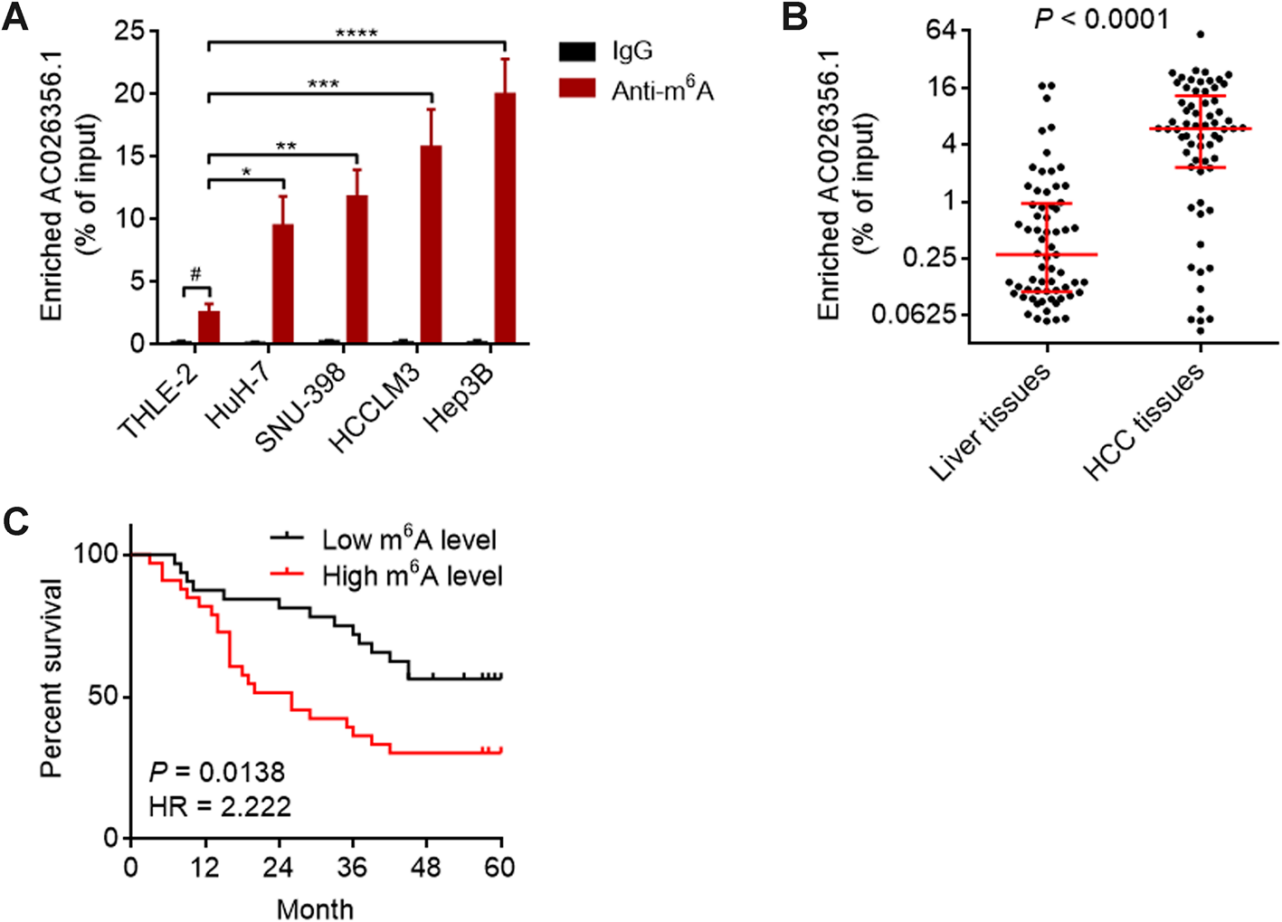
m^6^A modification level of AC026356.1 in HCC and its correlation with prognosis. (**A**) m^6^A modification levels of AC026356.1 in immortalized liver cell line THLE-2 and HCC cell lines HuH-7, SNU-398, HCCLM3, and Hep3B were detected using MeRIP-qPCR assays. Data are shown as mean ± SD of three independent experiments. ^#^*P* < 0.01 by Student’s *t*-test. *P < 0.05, **P < 0.01, ***P < 0.001, ****P < 0.0001 by one-way ANOVA followed by Dunnett's multiple comparisons test. (**B**) m^6^A modification levels of AC026356.1 in 65 pairs of HCC tissues and matched noncancerous liver tissues were detected using MeRIP-qPCR assays. Data are shown as median with interquartile range. *P* values were calculated using Wilcoxon matched-pairs signed rank test. (**C**) Kaplan–Meier analysis of the correlation between m^6^A modification level of AC026356.1 and overall survival in HCC (n = 65). Median m^6^A modification level of AC026356.1 was used as cut-off. HR and *P* values were calculated using log-rank test.

m^6^A modification levels of AC026356.1 in HCC tissues and cell lines were measured using MeRIP assays. The results showed that m^6^A-modified AC026356.1 was detected in all cell lines (Fig. 2A). However, the m^6^A modification level of AC026356.1 was significantly increased in HCC cell lines compared with that in immortalized liver cell line (Fig. 2A). The m^6^A modification level of AC026356.1 was also remarkably increased in HCC tissues compared with that in normal liver tissues (Fig. 2B). High m^6^A modification level of AC026356.1 in HCC was correlated with poor overall survival, which has a higher hazard ratio (HR) than the expression level of AC026356.1 (Fig. 2C, compared with Fig. 1H).

### m^6^A modification increases AC026356.1 transcript stability

**Figure 3:**
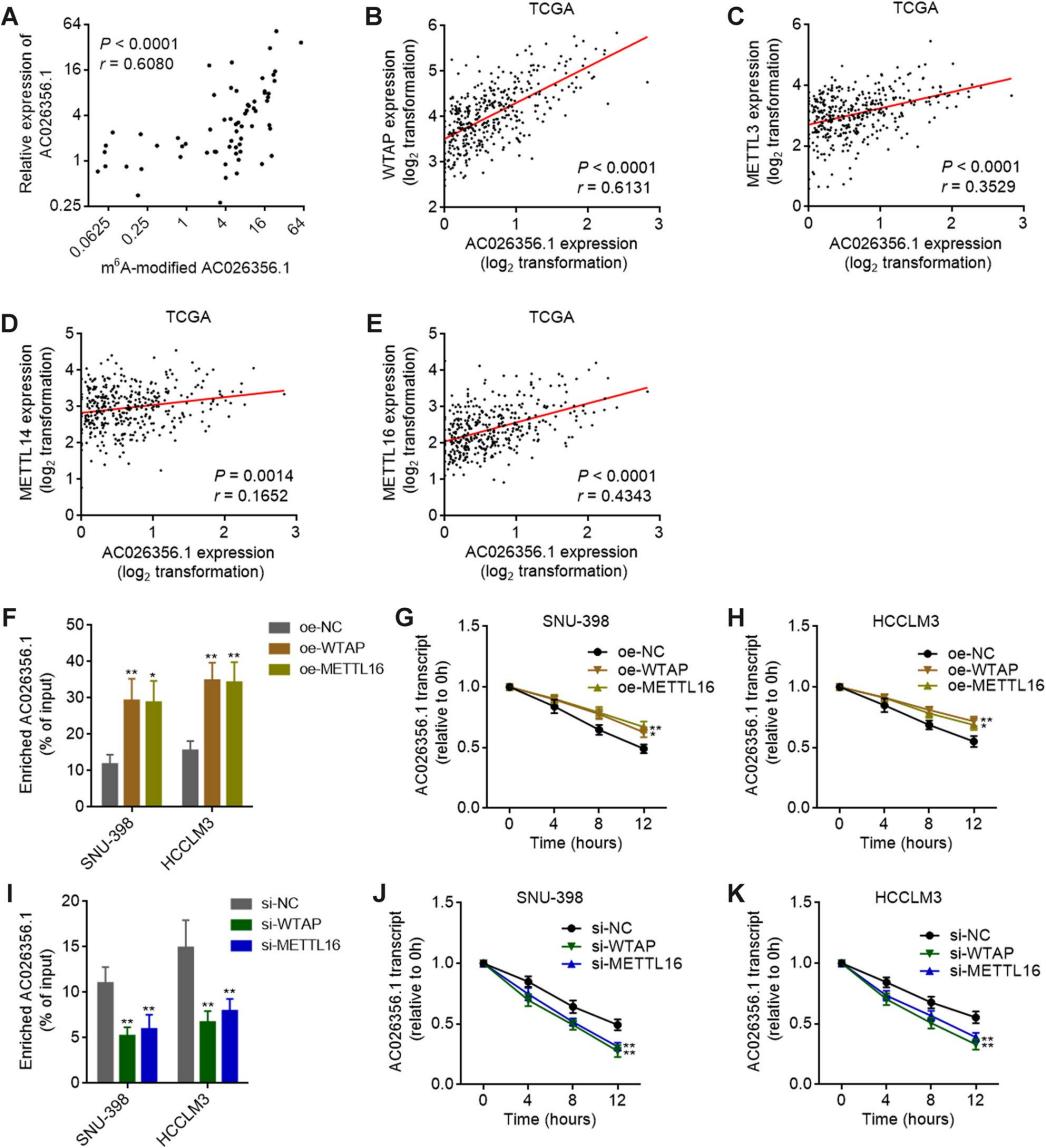
m^6^A modification increases AC026356.1 transcript stability. (**A**) The correlation between AC026356.1 expression level and m^6^A modification level of AC026356.1 in HCC tissues (n = 65). *P* and *r* values were calculated using Spearman correlation analysis. (**B**–**E**) The correlation between AC026356.1 expression level and WTAP (**B**), METTL3 (**C**), METTL14 (**D**), or METTL16 (**E**) expression levels in HCC tissues (n = 371) based on TCGA LIHC data. *P* and *r* values were calculated using Spearman correlation analysis. (**F**) m^6^A modification levels of AC026356.1 in SNU-398 and HCCLM3 cells with WTAP or METTL16 overexpression were detected using MeRIP-qPCR assays. (**G**,**H**) The stability of AC026356.1 transcript in SNU-398 (**G**) and HCCLM3 (**H**) cells with WTAP or METTL16 overexpression over time was detected after blocking new RNA synthesis with α-amanitin (50 µM) and normalized to 18S rRNA (a product of RNA polymerase I that is unchanged by α-amanitin). (**I**) m^6^A modification levels of AC026356.1 in SNU-398 and HCCLM3 cells with WTAP or METTL16 depletion were detected using MeRIP-qPCR assays. (**J**,**K**) The stability of AC026356.1 transcript in SNU-398 (**J**) and HCCLM3 (**K**) cells with WTAP or METTL16 depletion over time was detected after blocking new RNA synthesis with α-amanitin (50 µM) and normalized to 18S rRNA. For (**F**–**K**), data are shown as mean ± SD of three independent experiments. *P < 0.05, **P < 0.01 by one-way ANOVA followed by Dunnett's multiple comparisons test.

In our HCC cohort, the m^6^A modification level of AC026356.1 was positively correlated with the expression level of AC026356.1 in HCC tissues (Fig. 3A), implying that m^6^A modification may influence AC026356.1 expression. TCGA LIHC data revealed that the expression of AC026356.1 was positively correlated with methyltransferases WTAP, METTL3, METTL14, and METTL16 (Fig. 3B–E), supporting the potential positive regulation of AC026356.1 by m^6^A modification. Enhanced expression of WTAP or METTL16 upregulated m^6^A modification level of AC026356.1 in HCC cells (Fig. 3F), and increased AC026356.1 transcript stability (Fig. 3G,H). Depletion of WTAP or METTL16 reduced m^6^A modification level of AC026356.1 and decreased AC026356.1 transcript stability (Fig. 3I–K). These data suggested that m^6^A modification increases AC026356.1 transcript stability.

### Enhanced expression of AC026356.1 has oncogenic roles in HCC

**Figure 4:**
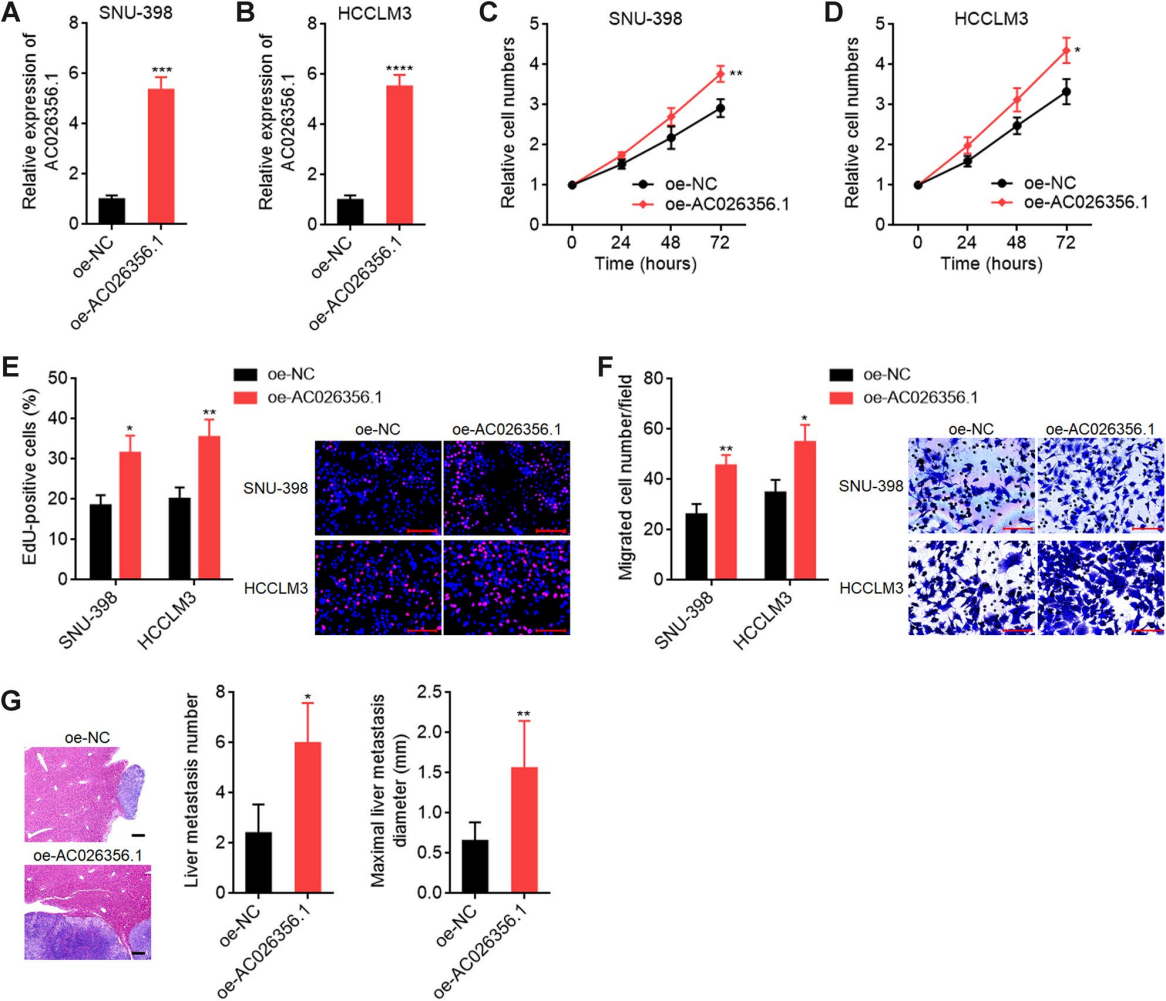
AC026356.1 has oncogenic roles in HCC. (**A**,**B**) AC026356.1 expression in SNU-398 (**A**) and HCCLM3 (**B**) cells with AC026356.1 stable overexpression was measured by qPCR. (**C**,**D**) Cell proliferation of SNU-398 (**C**) and HCCLM3 (**D**) cells with AC026356.1 overexpression was detected using CCK-8 assays. (**E**) Cell proliferation of SNU-398 and HCCLM3 cells with AC026356.1 overexpression was detected using EdU incorporation assay. Scale bars 100 µm. (**F**) Cell migration of SNU-398 and HCCLM3 cells with AC026356.1 overexpression was detected using transwell migration assay. Scale bars 100 µm. (**G**) Liver metastases were detected by HE staining 35 days after intrasplenic injection with indicated HCCLM3 cells. Data are shown as mean ± SD of three independent experiments (**A**–**F**) or n = 5 mice in each group (**G**). *P < 0.05, **P < 0.01, ***P < 0.001, ****P < 0.0001 by Student’s *t*-test (**A**–**F**) or Mann–Whitney test (**G**).

The potential roles of AC026356.1 in HCC were first investigated by stably overexpressing AC026356.1 in SNU-398 and HCCLM3 cells (Fig. 4A,B). CCK-8 assays showed that HCC cells with AC026356.1 overexpression had quicker cell proliferation than control cells (Fig. 4C,D). EdU incorporation assays further confirmed that overexpression of AC026356.1 promoted HCC cellular proliferation (Fig. 4E). Transwell migration assays showed that AC026356.1 overexpression promoted HCC cellular migration (Fig. 4F). Liver metastasis assays showed that HCCLM3 cells with AC026356.1 overexpression had more and larger liver metastatic tumors than control HCCLM3 cells (Fig. 4G).

### The oncogenic roles of AC026356.1 in HCC are dependent on m^6^A modification of AC026356.1

**Figure 5:**
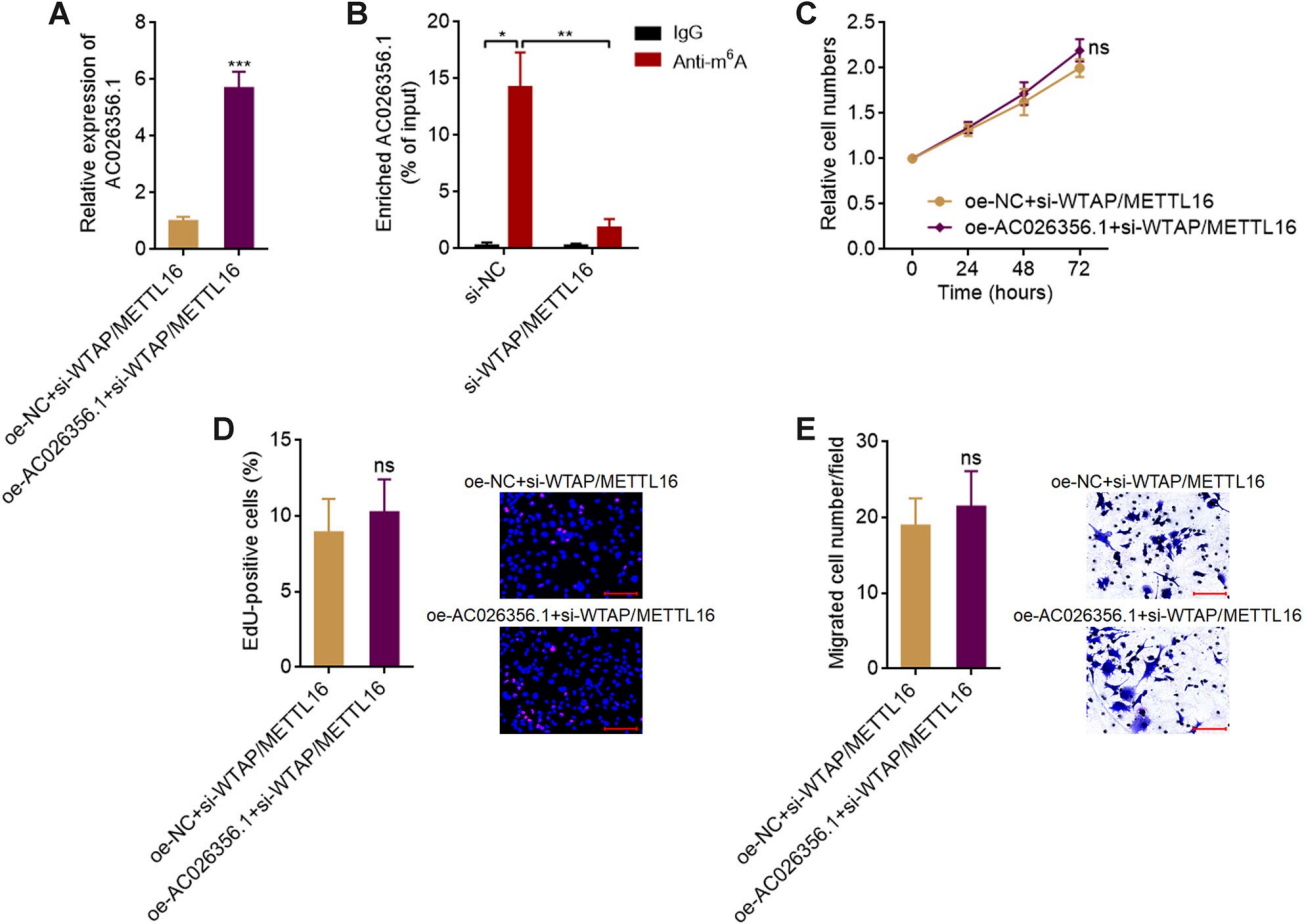
Loss of m^6^A modification abolished the oncogenic roles of AC026356.1 in HCC. (**A**) AC026356.1 expression in HCCLM3 cells with AC026356.1 overexpression and concurrent WTAP and METLL16 depletion was measured by qPCR. (**B**) m^6^A modification levels of AC026356.1 in HCCLM3 cells with WTAP and METTL16 depletion were detected using MeRIP-qPCR assays. (**C**) Cell proliferation of HCCLM3 cells with AC026356.1 overexpression and concurrent WTAP and METLL16 depletion was detected using CCK-8 assays. (**D**) Cell proliferation of HCCLM3 cells with AC026356.1 overexpression and concurrent WTAP and METLL16 depletion was detected using EdU incorporation assay. Scale bars 100 µm. (**E**) Cell migration of HCCLM3 cells with AC026356.1 overexpression and concurrent WTAP and METLL16 depletion was detected using transwell migration assay. Scale bars 100 µm. Data are shown as mean ± SD of three independent experiments. *P < 0.05, **P < 0.01, ***P < 0.001, ns, not significant, by Student’s *t*-test.

To investigate whether m^6^A modification contributes to the oncogenic roles of AC026356.1 in HCC, we knocked down WTAP and METTL16 in AC026356.1 overexpressed and control HCCLM3 cells, which extremely reduced the m^6^A modification level of AC026356.1 (Fig. 5A,B). At the condition of loss of m^6^A modification, overexpression of AC026356.1 did not have notable effects on cellular proliferation, as shown by CCK-8 and EdU incorporation assays (Fig. 5C,D). Transwell migration assays also showed that loss of m^6^A modification abolished the pro-migratory roles of AC026356.1 (Fig. 5E). These data suggested that the oncogenic roles of AC026356.1 are dependent on m^6^A modification of AC026356.1.

### AC026356.1 knockdown has tumor suppressive roles in HCC

To completely investigate the roles of AC026356.1, we stably silenced AC026356.1 in SNU-398 and HCCLM3 cells (Fig. S1A,B). CCK-8 and EdU incorporation assays showed that silencing of AC026356.1 repressed HCC cellular proliferation (Fig. S1C–E). Transwell migration assays showed that silencing of AC026356.1 repressed HCC cellular migration (Fig. S1F). These data suggested that AC026356.1 knockdown has tumor suppressive roles in HCC.

### AC026356.1 upregulates IL11 in an m^6^A-depedent manner

**Figure 6:**
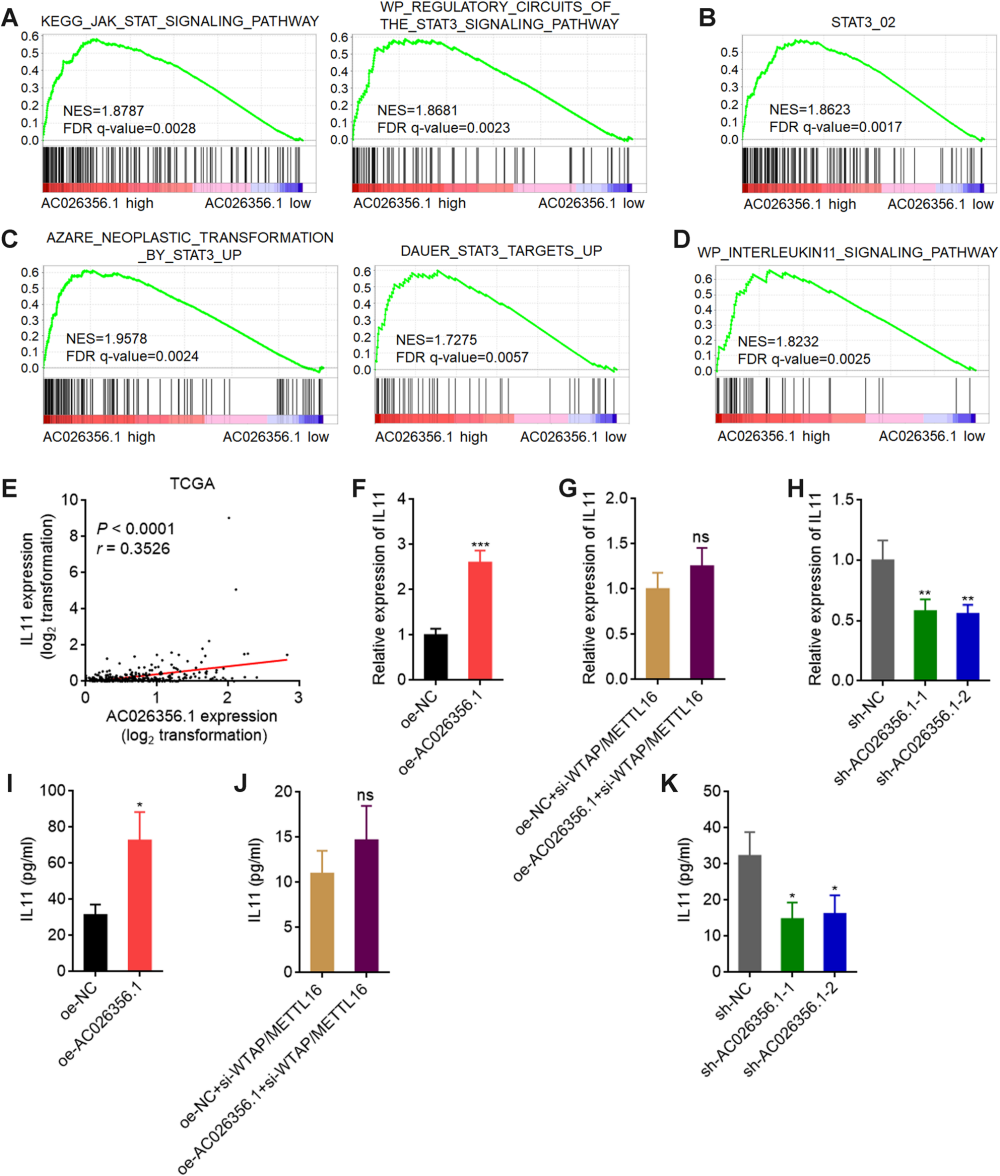
AC026356.1 upregulates IL11 in an m^6^A-depedent manner. (**A–D**) GSEA of KEGG JAK-STAT signaling pathway and WikiPathways STAT3 signaling pathway gene signatures (**A**), STAT3 transcription factor bound gene signature (**B**), STAT3-upregulated gene signatures (**C**), and WikiPathways IL11 signaling pathway gene signature (**D**) in AC026356.1 high expression group versus AC026356.1 low expression group. NES, normalized enrichment score. (**E**) The correlation between AC026356.1 and IL11 expression levels in HCC tissues (n = 371) based on TCGA LIHC data. *P* and *r* values were calculated using Spearman correlation analysis. (**F**) IL11 expression in HCCLM3 cells with AC026356.1 overexpression was measured by qPCR. (**G**) IL11 expression in HCCLM3 cells with AC026356.1 overexpression and concurrent WTAP and METLL16 depletion was measured by qPCR. (**H**) IL11 expression in HCCLM3 cells with AC026356.1 silencing was measured by qPCR. (**I**) IL-11 concentration in the culture medium of HCCLM3 cells with AC026356.1 overexpression was measured by ELISA. (**J**) IL-11 concentration in the culture medium of HCCLM3 cells with AC026356.1 overexpression and concurrent WTAP and METLL16 depletion was measured by ELISA. (**K**) IL-11 concentration in the culture medium of HCCLM3 cells with AC026356.1 silencing was measured by ELISA. For (**F**–**K**), data are shown as mean ± SD of three independent experiments. *P < 0.05, **P < 0.01, ***P < 0.001, ns, not significant by Student’s *t*-test (**F**,**G**,**I**,**J**) or one-way ANOVA followed by Dunnett's multiple comparisons test (**H**,**K**).

To investigate the mechanisms mediating the effects of AC026356.1, we performed Gene Set Enrichment Analysis (GSEA) using TCGA LIHC data. Median AC026356.1 expression level was used as cut-off to divide these HCC cases into AC026356.1 high or low expression groups. GSEA showed that STAT3 signaling pathways from KEGG and WikiPathways were both positively enriched in AC026356.1 high expression group (Fig. 6A). The genes whose promoter has STAT3 transcription factor binding sites were positively enriched in AC026356.1 high expression group (Fig. 6B). The genes upregulated by activated STAT3 were positively enriched in AC026356.1 high expression group (Fig. 6C). These data suggested that AC026356.1 activated STAT3 signaling in HCC. Furthermore, IL11 signaling pathway was also found to be positively enriched in AC026356.1 high expression group (Fig. 6D), further suggesting that AC026356.1 activated IL11/STAT3 signaling in HCC. TCGA LIHC data revealed that the expression of IL11 was positively correlated with AC026356.1 (Fig. 6E), supporting the regulation of IL11 by AC026356.1. IL11 mRNA expression was increased in HCCLM3 cells with AC026356.1 overexpression (Fig. 6F). The upregulation of IL11 mRNA by AC026356.1 was abolished by the loss of m^6^A modification (Fig. 6G). Silencing of AC026356.1 decreased IL11 mRNA expression (Fig. 6H). Protein expression level of IL11 was measured by ELISA. AC026356.1 overexpression increased IL11 concentration, which was abolished by the loss of m^6^A modification (Fig. 6I,J). Conversely, silencing of AC026356.1 decreased IL11 concentration (Fig. 6K). These data suggested that m^6^A-modified AC026356.1 upregulated IL11 and activated IL11/STAT3 signalling.

### m^6^A-modified AC026356.1 enhances IL11 mRNA stability via IGF2BP1

**Figure 7:**
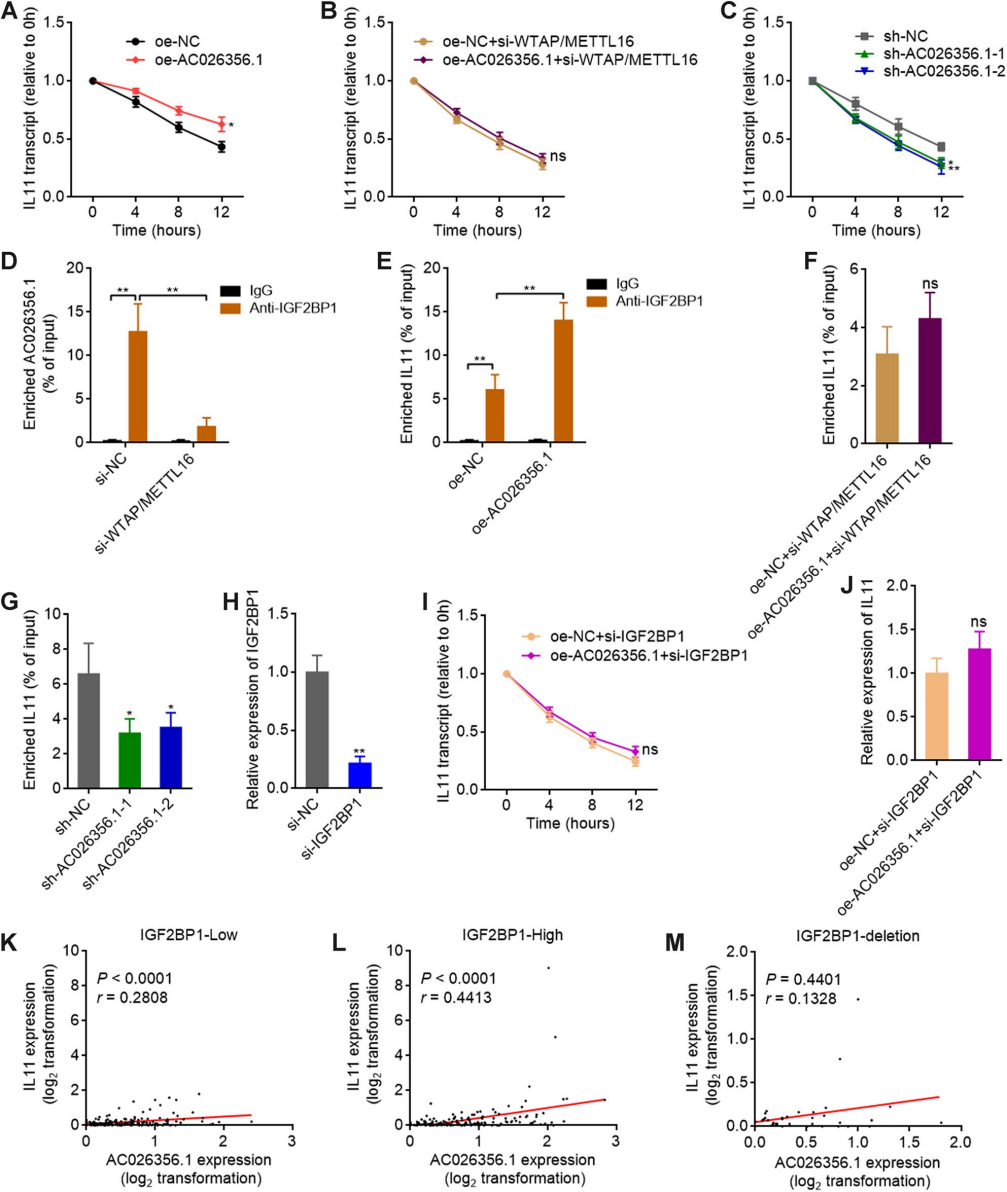
m^6^A-modified AC026356.1 enhances IL11 mRNA stability via IGF2BP1. (**A**) The stability of IL11 transcript in HCCLM3 cells with AC026356.1 overexpression over time was detected after blocking new RNA synthesis with α-amanitin (50 µM) and normalized to 18S rRNA. (**B**) The stability of IL11 transcript in HCCLM3 cells with AC026356.1 overexpression and concurrent WTAP and METLL16 depletion over time was detected after blocking new RNA synthesis with α-amanitin (50 µM) and normalized to 18S rRNA. (**C**) The stability of IL11 transcript in HCCLM3 cells with AC026356.1 silencing over time was detected after blocking new RNA synthesis with α-amanitin (50 µM) and normalized to 18S rRNA. (**D**) The binding of AC026356.1 to IGF2BP1 in HCCLM3 cells with WTAP and METTL16 depletion were detected using RIP-qPCR assays. (**E**) The binding of IL11 to IGF2BP1 in HCCLM3 cells with AC026356.1 overexpression were detected using RIP-qPCR assays. (**F**) The binding of IL11 to IGF2BP1 in HCCLM3 cells with AC026356.1 overexpression and concurrent WTAP and METLL16 depletion were detected using RIP-qPCR assays. (**G**) The binding of IL11 to IGF2BP1 in HCCLM3 cells with AC026356.1 silencing were detected using RIP-qPCR assays. (**H**) IGF2BP1 expression in HCCLM3 cells with IGF2BP1 depletion was measured by qPCR. (**I**) The stability of IL11 transcript in HCCLM3 cells with AC026356.1 overexpression and concurrent IGF2BP1 depletion over time was detected after blocking new RNA synthesis with α-amanitin (50 µM) and normalized to 18S rRNA. (**J**) IL11 expression in HCCLM3 cells with AC026356.1 overexpression and concurrent IGF2BP1 depletion was measured by qPCR. For (**A**–**J**), data are shown as mean ± SD of three independent experiments. *P < 0.05, **P < 0.01, ns, not significant by Student’s *t*-test (**A**,**B**,**D**–**F**,**H**–**J**) or one-way ANOVA followed by Dunnett's multiple comparisons test (**C**,**G**). (**K**,**L**) The correlation between AC026356.1 and IL11 expression levels in IGF2BP1 lowly expressed (n = 186) (**K**) or IGF2BP1 highly expressed HCC tissues (n = 185) (**L**) based on TCGA LIHC data. (**M**) The correlation between AC026356.1 and IL11 expression levels in IGF2BP1 depleted HCC tissues (n = 36) based on TCGA LIHC data. For (**K–M**), *P* and *r* values were calculated using Spearman correlation analysis.

To analyze whether AC026356.1 modulates IL11 mRNA generation or degradation, we treated AC026356.1 overexpressed and control HCCLM3 cells with α-amanitin to block new RNA synthesis, and then detected the degradation of IL11 mRNA. The results showed that overexpression of AC026356.1 increased IL11 transcript stability (Fig. 7A). At the condition of m^6^A loss, AC026356.1 did not have notable effect on IL11 transcript stability (Fig. 7B). Silencing of AC026356.1 decreased IL11 transcript stability (Fig. 7C). These data suggested that m^6^A-modified AC026356.1 increased IL11 transcript stability. The online tool RM2Target (http://rm2target.canceromics.org/) predicted the m^6^A reader IGF2BP1 as a binding partner of AC026356.1. RIP assays showed that IGF2BP1 specifically bound to AC026356.1, which was extremely reduced by the loss of m^6^A modification (Fig. 7D). These data suggested that IGF2BP1 binds m^6^A-modified AC026356.1. IGF2BP1 has been frequently reported to bind and stabilize target mRNAs^44,45^. Thus, we further investigated whether IGF2BP1 regulates IL11 transcript stability and whether m^6^A-modified AC026356.1 influences this effect. RIP assays showed that IGF2BP1 interacted with IL11 transcript (Fig. 7E). The binding of IGF2BP1 to IL11 transcript was increased by overexpression of AC026356.1 (Fig. 7E). Loss of m^6^A modification abolished the increased interaction between IGF2BP1 and IL11 transcript caused by AC026356.1 overexpression (Fig. 7F). Conversely, AC026356.1 silencing reduced the binding of IGF2BP1 to IL11 transcript (Fig. 7G). These data suggested that m^6^A-modified AC026356.1 bound to IGF2BP1, which promoted the interaction between IGF2BP1 and IL11 transcript. To investigate whether IGF2BP1 mediates the roles of AC026356.1 in increasing IL11 transcript stability, we depleted IGF2BP1 expression in HCCLM3 cells (Fig. 7H). RNA degradation assays showed that depletion of IGF2BP1 abolished the increased IL11 transcript stability caused by AC026356.1 overexpression (Fig. 7I, compared with Fig. 7A). Similarly, depletion of IGF2BP1 also abolished the increased IL11 expression caused by AC026356.1 overexpression (Fig. 7J, compared with Fig. 6F). TCGA LIHC data showed that HCC tissues with high IGF2BP1 expression had a higher correlation coefficient between IL11 and AC026356.1 expression (*r* = 0.4413) than those HCC tissues with low IGF2BP1 expression (*r* = 0.2808) (Fig. 7K,L). TCGA LIHC data further showed that at the condition of IGF2BP1 depletion, the expression of IL11 was not correlated with AC026356.1 (Fig. 7M), supporting that IGF2BP1 is necessary for the effects of m^6^A-modified AC026356.1 on IL11.

### Depletion of IL11 attenuates the oncogenic roles of AC026356.1 in HCC

To assess whether IL11 is the mediator of the oncogenic roles of AC026356.1 in HCC, we silenced IL11 expression in AC026356.1 overexpressed HCCLM3 cells (Fig. S2A). CCK-8 and EdU incorporation assays showed that silencing of IL11 largely reversed the pro-proliferative roles of AC026356.1 (Fig. S2B,C). Transwell migration assays showed that silencing of IL11 also largely reversed the pro-migratory roles of AC026356.1 (Fig. S2D). These data suggested that IL11 is a critical mediator of the oncogenic roles of AC026356.1 in HCC.

## Discussion

AC026356.1 has 1357 nucleotides in length and only one exon. *AC026356.1* is located in chromosome 12p11.21. Previous reports found that AC026356.1 was upregulated and correlated with poor overall survival in lung adenocarcinoma^46^. m^6^A modification induced upregulation of AC026356.1, which promoted proliferation, migration, and stemness of lung adenocarcinoma cells^47^. Here, we further identified AC026356.1 as an m^6^A-modified oncogenic lncRNA in HCC. Not only TCGA LIHC data, but also our own HCC cohort, revealed that the expression of AC026356.1 is increased in HCC and high expression of AC026356.1 is correlated with poor overall survival of HCC patients. More importantly, we found that the m^6^A modification level of AC026356.1 is increased in HCC tissues and cell lines. Increase m^6^A modification level of AC026356.1 is correlated with poor overall survival of HCC patients. Thus, our findings suggested that aberrant m^6^A modification is involved in HCC and may be used as prognostic biomarker for HCC.

Consistent with the previous report about the role of m^6^A modification in AC026356.1 expression in lung adenocarcinoma^47^, we also found that m^6^A modification increased AC026356.1 transcript stability in HCC. Consistent with the reported oncogenic roles of AC026356.1 in lung adenocarcinoma^47^, we also found that AC026356.1 promoted HCC cellular proliferation, migration, and liver metastasis in vivo. However, our findings further showed that the oncogenic roles of AC026356.1 in HCC are dependent on m^6^A modification. At the condition of m^6^A modification remove by depletion of WTAP and METTL16, ectopic expression of non-m^6^A-modified AC026356.1 did not have notable effects on HCC cell. Thus, our findings suggested that m^6^A modification not only regulates AC026356.1 expression, but also influences the function of AC026356.1.

Mechanistic investigations revealed that m^6^A-modified AC026356.1 activated IL11/STAT3 signaling pathway through upregulating IL11 expression in HCC. The positive roles of AC026356.1 in regulating IL11/STAT3 are also dependent on m^6^A modification of AC026356.1. Further elucidating the mechanisms underlying the regulation of IL11 by m^6^A-modified AC026356.1, we identified the m^6^A reader IGF2BP1 specifically bound to m^6^A-modified AC026356.1. The interacting between IGF2BP1 and m^6^A-modified AC026356.1 further promoted the binding between IGF2BP1 and IL11 mRNA. IGF2BP1 is a conserved oncofetal protein, which has oncogenic roles in various malignancies^44^. The main mechanism of IGF2BP1 is to stabilize the RNAs bound by IGF2BP1^45^. Here, we identified IL11 mRNA as a target of IGF2BP1. Moreover, we found that m^6^A-modified AC026356.1 enhanced the roles of IGF2BP1 in stabling IL11 mRNA. The potential influences of m^6^A-modified AC026356.1 on other targets of IGF2BP1 need further investigation. Nevertheless, functional rescue assays showed that depletion of IL11 reversed the oncogenic roles of AC026356.1 in HCC, supporting IL11 as a critical downstream target of AC026356.1.

## Conclusion

The m^6^A modification level of AC026356.1 is increased in HCC and high m^6^A modification level of AC026356.1 is associated with poor survival of HCC patients. m^6^A-modified AC026356.1 plays oncogenic roles in HCC via interacting with IGF2BP1 and enhancing IL11 mRNA stability, which leads to the activation of IL11/STAT3 signaling. This study suggested that m^6^A-modified AC026356.1 may be a prognostic biomarker and therapeutic target for HCC.

### Supplementary Information

Supplementary Figure 1.

Supplementary Figure 2.

Supplementary Legends.

## Data Availability

The datasets generated and/or analyzed during the current study are available from the corresponding author on reasonable request.
