# Supplementary figures and images for: m6A modification of AC026356.1 facilitates hepatocellular carcinoma progression by regulating the IGF2BP1-IL11 axis
Source: Sci Rep. 2023 Nov 5;13:19124. doi: 10.1038/s41598-023-45449-w (PMC10625930; doi:10.1038/s41598-023-45449-w)

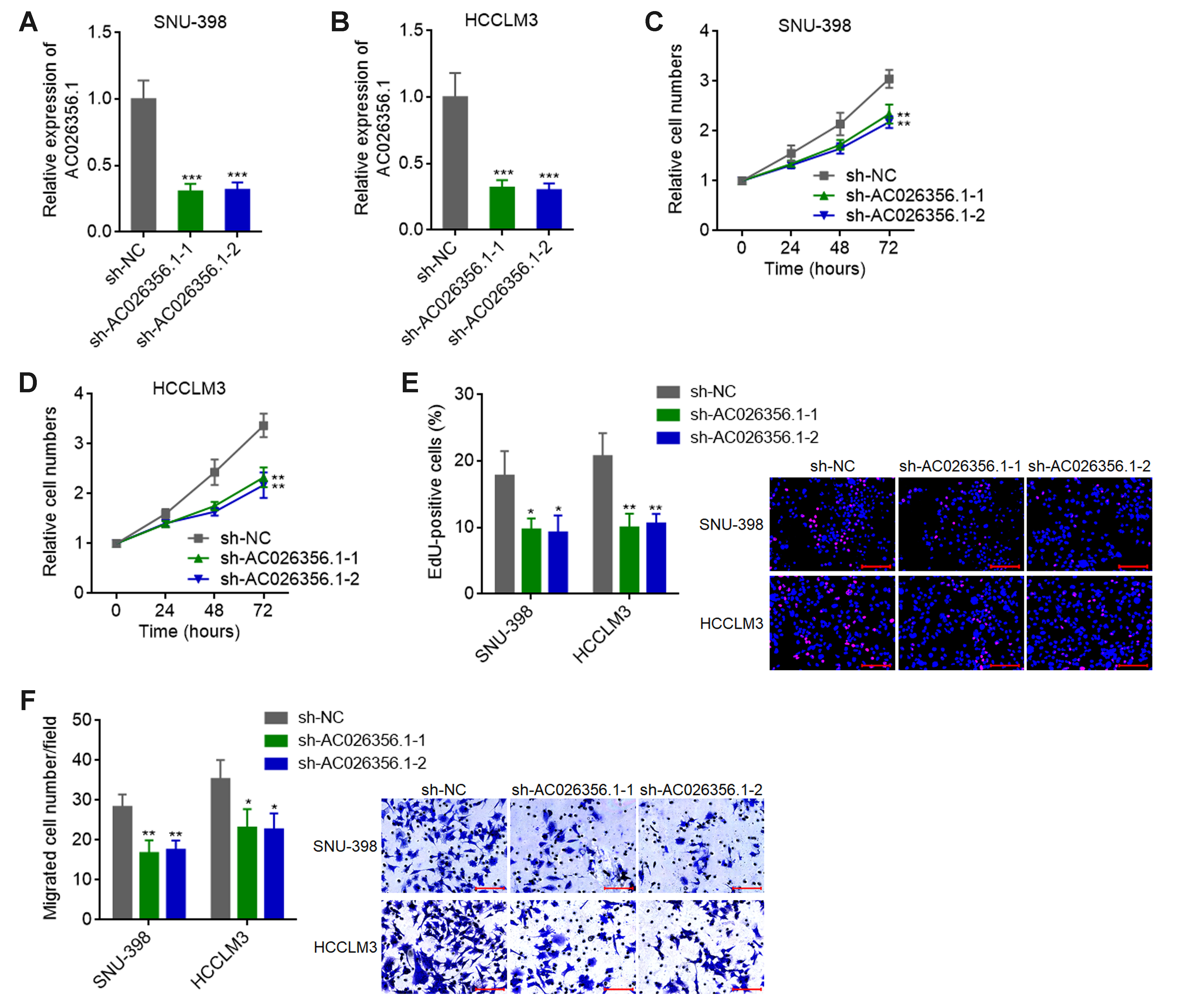

Supplement: Supplementary file 1 — Supplementary Figure 1. [file 41598_2023_45449_MOESM1_ESM.tif]

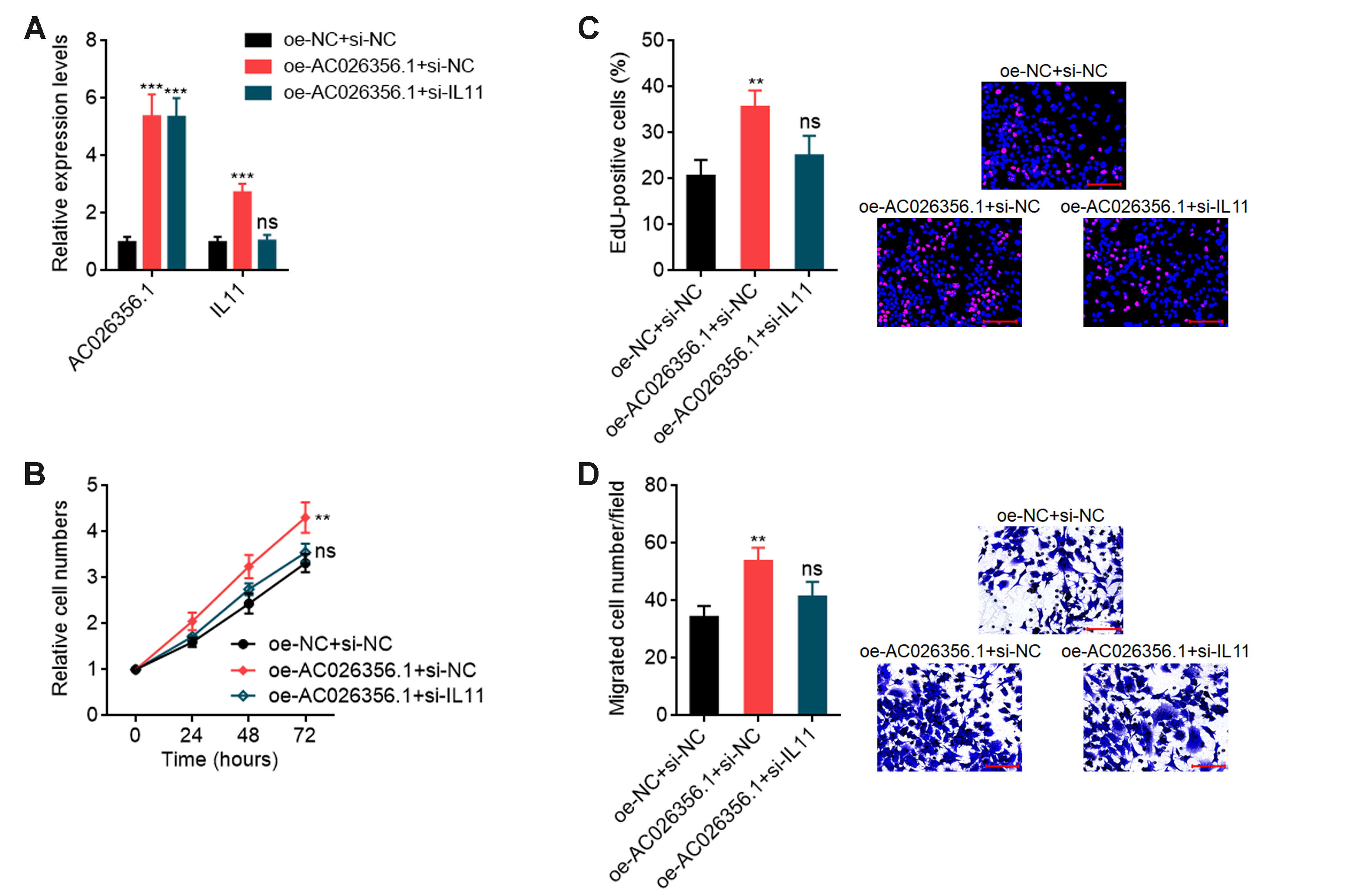

Supplement: Supplementary file 2 — Supplementary Figure 2. [file 41598_2023_45449_MOESM2_ESM.tif]
