# Supplementary material for: m6A modification of AC026356.1 facilitates hepatocellular carcinoma progression by regulating the IGF2BP1-IL11 axis
Source: Sci Rep. 2023 Nov 5;13:19124. doi: 10.1038/s41598-023-45449-w (PMC10625930; doi:10.1038/s41598-023-45449-w)
Supplement: Supplementary file 3 — Supplementary Legends. [file 41598_2023_45449_MOESM3_ESM.docx]

**Supplementary figure legends**

**Figure S1. AC026356.1 silencing has tumor suppressive roles in HCC. A**, **B** AC026356.1 expression in SNU-398 (**A**) and HCCLM3 (**B**) cells with AC026356.1 stable silencing was measured by qPCR. **C**, **D** Cell proliferation of SNU-398 (**C**) and HCCLM3 (**D**) cells with AC026356.1 silencing was detected using CCK-8 assays. **E** Cell proliferation of SNU-398 and HCCLM3 cells with AC026356.1 silencing was detected using EdU incorporation assay. Scale bars = 100 µm. **F** Cell migration of SNU-398 and HCCLM3 cells with AC026356.1 silencing was detected using transwell migration assay. Scale bars = 100 µm. Data are shown as mean ± SD of three independent experiments. **P* < 0.05, ***P* < 0.01, ****P* < 0.001 by one-way ANOVA followed by Dunnett's multiple comparisons test.

**Figure S2. Depletion of IL11 attenuates the oncogenic roles of AC026356.1 in HCC. A** AC026356.1 and IL11 expression levels in HCCLM3 cells with AC026356.1 overexpression and concurrent IL11 depletion were measured by qPCR. **B** Cell proliferation of HCCLM3 cells with AC026356.1 overexpression and concurrent IL11 depletion was detected using CCK-8 assays. **C** Cell proliferation of HCCLM3 cells with AC026356.1 overexpression and concurrent IL11 depletion was detected using EdU incorporation assay. Scale bars = 100 µm. **D** Cell migration of HCCLM3 cells with AC026356.1 overexpression and concurrent IL11 depletion was detected using transwell migration assay. Scale bars = 100 µm. Data are shown as mean ± SD of three independent experiments. ***P* < 0.01, ****P* < 0.001, ns, not significant, by one-way ANOVA followed by Dunnett's multiple comparisons test.
